# Harnessing local traditional authorities as a potential strategy to combat the vagaries of climate change in Zimbabwe

**DOI:** 10.4102/jamba.v10i1.651

**Published:** 2018-09-25

**Authors:** Happwell Musarandega, Wisemen Chingombe, Rajendran Pillay

**Affiliations:** 1 Department of Geography and Environmental Science, University of Fort Hare, South Africa; 2 School of Biology and Environmental Sciences, University of Mpumalanga, South Africa

## Abstract

While the devastating vagaries of climate change are ravaging communities the world over, especially in Africa, and Zimbabwe in particular, the role of traditional authorities is being overlooked. This paper argues for a relentless push towards the unimpeded involvement of local traditional authorities (LTAs) in the mobilisation of rural communities to adopt appropriate climate change adaptation practices in Zimbabwe. Given its complexity and uniqueness, external intervention through government and non-governmental agents alone can hardly foster climate change adaptation particularly at local levels within communities. Traditional leaders, who have for a long time been useful in the governance of people in various rural communities, can play a supportive role in climate change adaptation. Traditional leaders do not only serve as governance authorities but also know the traditional strategies of combating the negative effects of climate change. Despite the pressure from political interference and the advent of western technological advancement, a lot could still be done to buttress the authority and respect vested in chiefs, headsmen and village heads in the country. LTAs have the power to manage grassroots communities; hence they can be utilised as drivers in the use of traditional climate change adaptation strategies. The paper concludes that political interference is one challenge faced by abusing traditional leadership as a means to gain political mileage. The paper recommends for extended capacity building on the part of traditional leaders to improve their knowledge base. This will enable them to appreciate the integration of indigenous and modern climate change adaptation strategies. It further recommends the revitalisation of the traditional council (Dare raMambo) to deal with environmental offenses with the scope of assisting government efforts to ensure sound ecological practices within communities.

## Introduction

Zimbabwe is a developing country with the majority of its people living in rural community setups. As such, agriculture remains the chief livelihood practice and contributes up to 70% of the country’s revenue (Moyo et al. 2012; Shoko & Shoko 2013). Smallholder farmers make up the majority of agricultural producers (Mavhura, Manatsa & Mushore 2015). Of these, the majority depends mainly on rain-fed agriculture, a scenario that renders them vulnerable to the negative impact of climate change (Mavhura, Manatsa & Matiashe 2017). The Inter-governmental Panel on Climate Change (IPCC 2014) provided a definition of climate change, which reads as follows:

Climate change refers to a change in the state of the climate that can be identified (e.g., by using statistical tastes) by changes in the mean and/or the variability of its properties and that persists for an extended period, typically decades or longer. Climate change may be due to natural internal processes or external forcings such as modulations of solar cycles, volcanic eruptions, and persistent anthropogenic changes in the composition of the atmosphere or in land use. (p. 5)

Therefore, climate change is a phenomenon whose change indicators can be quantifiably deduced and whose causes are broadly understood to be both human and naturally triggered.

Issues of appropriate governance policy play a significant role in the wake of climate change. Governance refers to the processes, structures and organisational traditions that determine how power is exercised. Included within the concept is the idea how stakeholders have their say, how decisions are taken and how decision makers are held to account. In Zimbabwe, the traditional authorities are empowered by an act of parliament to ensure traditional climate change adaptation strategies are applied. The *Traditional Leaders Act* Chapter 29:17, Sections 5, 9 and 12 spell out the duties of traditional leaders at their different levels, to ensure that the inhabitants are protected from natural disasters. Marango et al. (2016b) posited that considering the fact that climate change causes water and food insecurity, there is a need to build an understanding of how indigenous and scientific knowledge systems can be integrated to combat this problem. Climate change is a natural disaster that attracted the attention of all government. Sustainable Development Goal (SDG) 13 is a stand-alone goal on climate change. It instructs all governments to *Take urgent action to combat climate change and its impacts*.

Widespread discussions with regard to climate change causes and mitigation have been conducted at world forums facilitated by the United Nations Framework Convention on Climate Change (UNFCCC) since Rio 1992 (Manyanhaire & Chitura 2015). Examples of such interventions include the Kyoto Protocol (2008) meant to compel industrialised countries to reduce greenhouse gas (GHG) emissions by an average of 5% against 1990 levels over the 5-year period 2008–2012 (UNFCC 2007). Most of such large-scale initiatives and policy frameworks end up with limited answers in terms of explaining the actual impact of climate change on the socio-economic dynamics within communities in a country like Zimbabwe (Manyanhaire & Chitura 2015). This signals for more localised and home-grown initiatives that suit the uniqueness of individual community settings in the country.

Based on the UN SDG 13, there is need for some investment on climate change adaptation. However, this calls for huge investment funds as well as a great deal of expertise. In order to build more adaptive capacity, developing countries need to receive more development aid from rich countries (Chambwera & Stage 2010). The IPCC (2014:72) defined climate change adaptation as ‘the process of adjustment to actual or expected climate and its effects’ and further added that ‘in human systems, adaptation seeks to moderate harm or exploit beneficial opportunities’. Most climate change adaptation initiatives in the developing world have therefore been championed by non-governmental organisations that have the funding capacity to do so. However, the tendency by the developing world to rely on donor-funded programmes seems to be a fairly risky syndrome because most of their programmes are small-scale and short-term interventions instead of long-term transformations (Atyang & Standley 2014).

Based on the foregoing argument, applying a complementarity of both traditional and locally oriented solutions with the aid of by external support would suffice a lot. Marango et al. (2016a) postulated that failure of co-existence of scientific and indigenous knowledge (IK) led to the current level of the negative effects of climate change. This is so because fighting climate change from an external and broader global perspective alone remains a daunting task at a local level. Instead, valuing local adaptive beliefs and practices complemented by external perspectives provides a working solution to tackle the deadly climate change phenomenon. It is important to note that traditional climate change adaptation strategies are more compatible with local realities. This is so because livelihoods differ from one setting to another (Mavhura 2017). Important to note is the fact that the impacts of climate change are equally felt at different levels within country settings (Dube & Phiri 2013). Against this background, the aim of this paper is to explore the role of traditional authorities, in particular their potential as climate change adaptation strategists. Traditional leaders are recognised as ‘strategic agents of change at the community level’ (Baldwin & Muyengwa 2014).

## Structure of traditional leadership in Zimbabwe

Local traditional authorities (LTAs) in Zimbabwe fall under the Ministry of Local Government, Public Works and National Housing. It is the responsibility of the ministry to administer all Acts of Parliament and regulatory instruments designed to govern the operations of LTAs. Traditional leadership is hierarchical, involving three levels as according to Baldwin and Muyengwa (2014).

The highest level is that of a chief [*mambo*], followed by a headman [*sadunhu*] in the middle and a village head [*sabhuku*] at the lowest level of the rank. Chiefs are appointed by the president of the country (Makumbe 2010). Succession with regard to traditional leadership is on the hereditary basis as opposed to elected local government structures that are instituted on the basis of Acts of Parliament. In the section that follows, the author introduces to the readers the role of LTAs as the custodians of local, traditional or IK.

## Local traditional authorities as custodians of indigenous knowledge

LTAs play a big role in ensuring that traditions are respected and maintained for the benefit of their posterity. They are the custodians of indigenous knowledge systems (IKS) and its related technologies. IKSs is defined as a systematic body of information. It is the totality of information, skills and practices acquired by local people through past experiences, observations, informal experiments and intimate understanding of their environment (Marango 2011; Marango et al. 2016b). To date, several rural people within the smallholder farmer community still bear little access and interpretive skills to make full use of modern weather forecasts transmitted through print and electronic media (Shoko & Shoko 2013). In the backdrop of this complexity, such people are left with no choice except to utilise their easily comprehensible IK. In fact, long before the subject of climate change came into limelight, rural people used to experience adverse weather conditions. Indigenous forms of knowledge were therefore long been used to explain incidences of meteorological phenomena such as drought, storms and floods, thereby guiding local communities (Nakashima et al. 2012). Emeagwali (2014) defines IK as that which is generated by people in a particular societal setting. Previously, Nakashima et al. (2012) had indicated that IK implies knowledge and expertise amassed through generations and transformed by each successive generation, which direct human societies in their interaction with their immediate environment. IK covers different aspects of human life, which include climate, agriculture, linguistics, animal husbandry and botany (Mapira & Mazambara 2013).

**FIGURE 1:**
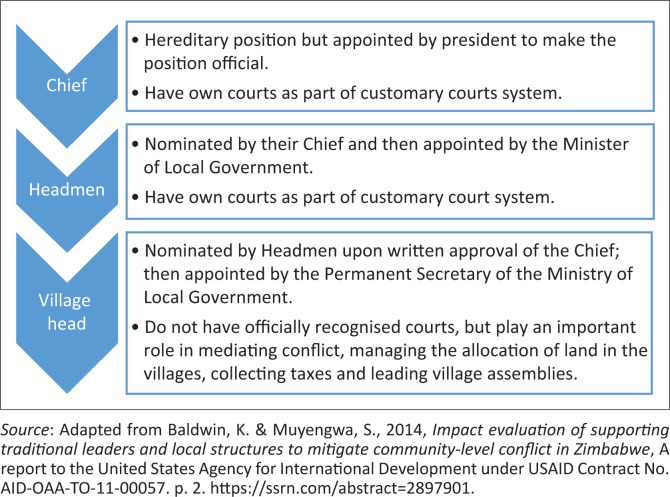
Hierarchy of traditional leadership in Zimbabwe.

Literature proves beyond doubt the fact that LTAs are custodians of IK within communities (Mapara 2009). Traditional practices and knowledge have, for long, been used to guide people in their quest to live harmoniously with their environment even before the advent of western forms of knowledge (Makumbe 2010; Manyanhaire & Chitura 2015; Shoko & Shoko 2013). Locally designed forms of knowledge were used to help people to respond to these weather-related developments. Thus, community resources such as land and water have always been administered using traditional forms of governance under the guidance of traditional leaders. A study on the perceptions of farmers on climate change in Masvingo Province revealed that about 48% of farmers felt that weather changes were largely a result of digression from cultural and religious factors (Moyo et al. 2012). Dube and Phiri (2013) also noted that farmers in Matobo believed that the aridity phenomenon in the area could be answered best by obeying to the gods.

The *Zunde raMambo* (‘the chief’s granary’) is a typical scheme used in developing self-sustenance of communities and decreasing the vulnerability of local people to food shortage. The local chief provides land for the *Zunde raMambo* project while community members render their labour services to the scheme through a collective action approach (Mavhura 2017). The proceeds from the scheme target most vulnerable members of the community such as orphans, widows, elderly and sick people. In a study of rural communities in the Zambezi valley of Zimbabwe, Mavhura (2017) established that informal safety nets, collective work and share-rearing arrangement of draught power enhance resilience to food insecurity in rural communities. In fact, these were notably more successful under the traditional institutions’ guidance.

In Zimbabwe, many sacred forests, mountains and river courses exist in which LTAs are known to have control over. This is enshrined in the *Traditional Leaders Act*. Such heritage sites form vital ecological corridors in the country such that LTAs’ control over these, therefore, becomes a necessary prerequisite for sound ecological management in the wake of climate change. Marango (2017) affirmed the importance of traditional leadership in custodianship of natural resources as a strategy for combating the negative effects of climate change. Literature has it that in some communities, developmental undertakings have stalled following the alleged breaching of certain cultural norms and values (Gordon 2006). Under such circumstances, LTAs were often called in to intervene by harmonising mysteriously conflicting situations through ritual performances at affected sites. In this regard, LTAs have the potential to call off certain developmental initiatives, which they consider to be violating the environmental stability of certain places.

## International recognition for traditional leadership

On a global scale, IK has gained more recognition after the United Nations Conference on Environment and Development, which was held in Rio de Janeiro, Brazil, in June 1992. Article 8(j) in Secretariat of the Convention on Biological Diversity (2005) clearly states that:

Each Contracting Party shall, as far as possible and as appropriate

(j) Subject to its national legislation, respect, preserve and maintain knowledge, innovations and practices of indigenous and local communities embodying traditional lifestyles relevant for the conservation and sustainable use of biological diversity and promote their wider application with the approval and involvement of the holders of such knowledge, innovations and practices and encourage the equitable sharing of the benefits arising from the utilisation of such knowledge, innovations and practices. (p.138)

The recognition of IK whose custodians are LTAs (Mapara 2009; Mapira & Mazambara 2013; Shoko & Shoko 2013) was accredited in the Fourth Assessment Report of the IPCC as ‘an invaluable basis for developing adaptation and natural resource management strategies in response to environmental and other forms of change’ (IPCC 2007 in Nakashima et al. 2012:24). The gratitude to traditional values was reaffirmed at IPCC’s 32nd Session as a guiding principle for the Cancun Adaptation Framework adopted by Parties at the 2010 UNFCCC Conference in Cancun (Nakashima et al. 2012:24).

Given the greater involvement of humanitarian organisations within communities, some people have become increasingly detached from the usual traditional governance and align with modern ways. This is so because the so-called development agencies bring blueprints without considering the local as if they are *tabla rasas* (Marango 2011). When community-based programmes are introduced within communities, they are usually run on the basis of some legal structure guided by some constitution. When communities move away from traditional governance to legal statutes, it means there is a need for personnel such as the police to ensure that the constitution set is followed and to apprehend those who breach the set mandates. When LTAs try to meddle in matters where a legal constitution is in place, their presence is not seriously felt because the constitution reduces everyone to the same level and leaves no cut above the rest. In the administration of community practices, police officials are often too detached from communities to effectively oversee the daily goings on and this is the gap that LTAs are needed to fill, because they live with the people.

## Local traditional authorities as constitutionally mandated leaders in Zimbabwe

LTAs have worked with successive government regimes even before independence ensuring that there was compliance between communities and governing authorities (Makumbe 2010). In fact, Zimbabwe is a signatory to the UN Convention on Biological Diversity (CBD). Since the ratification of the CBD, the government has given more empowerment to LTAs to exercise their mandate over the communities in which they administer. Chiefs and headsmen have the authority to give land to people in rural areas for settlement and agricultural purposes (Baldwin & Muyengwa 2014; Makumbe 2010). The only exception to this power is the provision of land to business people in areas designated for business purposes such as rural service centres and growth points. Unfortunately, most of them have knowledge gaps, which make them fail to realise the rights and powers that they have (Baldwin & Muyengwa 2014).

Environmental issues such as overgrazing, over cultivation and deforestation were to be suppressed by LTAs through the new powers bestowed in them (Madondo 2000, cited in Ncube 2011). The renewed *Traditional Leaders Act (2000)* added more powers to LTAs by mainstreaming them into planning and community development initiatives (Ncube 2011). The Constitution of Zimbabwe accords chiefs, headmen and village heads Presidential appointee status and therefore gives them the power to, among other things, uphold cultural values and control the use of land and natural resources in line with government legislative frameworks. According to Government of Zimbabwe (2013), *Constitution of Zimbabwe Amendment (Number 20) Act 2013* Chapter 15 section 282 (1) clearly states the responsibilities of traditional leaders, which are:

- To promote and uphold the cultural values of their communities and, in particular, to promote sound family values.
- To take measures to preserve the culture, traditions, history and heritage of their communities, including sacred shrines.
- To facilitate development; In accordance with an Act of Parliament, to administer communal land and to protect the environment.
- In accordance with Act of Parliament, to administer Communal Land and to protect the environment.
- To resolve disputes amongst people in their communities in accordance with customary law.
- To exercise any other functions conferred or imposed on them by an Act of Parliament.

With all these powers to uphold cultural values, preserve heritage sites within communal lands, Chiefs, headsmen and village heads have the potential to protect forests, water courses and other ecologically valuable entities. These leaders have the potential to make key decisions with regard to where their subjects settle, what they do and how they do it. This is a vital position for them to ensure that human practices within areas of their jurisdiction are sustainable and in line with climate change adaptation demands.

Research has shown that there is some increasingly serious political interference in the country which detracts the efforts of LTAs in their quest to deliver their mandate (Baldwin & Muyengwa 2014). For instance, the government’s 2000 fast track resettlement programme was done on political grounds putting little consideration for environmental concerns (Makumbe 2010). Issues with regard to frequent fire outbreaks particularly after the year 2000 have been linked to the resettlement programme (Manyanhaire & Chitura 2015). Despite the heavy political interference behind the resettlement programme, LTAs, given their constitutional recognition, still have the potential to wade off acts of external interference. The *Constitution of Zimbabwe Amendment (Number 20) Act 2013* Chapter 15 section 281 (2) rightly states that traditional leaders must not:

- be members of any political party or in any way participate in partisan politics
- act in a partisan manner
- further the interest of any political party
- violate the fundamental rights and freedoms of any person.

In the wake of their constitutional cover, local traditional leaders have the latitude to work in liaison with agents such as Environmental Management Agency (EMA), Forestry Commission and Zimbabwe Republic Police to protect the environment from damage. In fact, EMA in its operational framework does recognise the role of LTAs. This platform, therefore, forms a formidable taskforce capable of ensuring that sustainable practices are put in place within rural communities.

## Local traditional authorities as potential drivers of climate change adaptation

Environmental challenges, including weather uncertainty, have long confronted humanity within various community settings and traditional forms of knowledge have played a significant role in countering the challenges using their local knowledge base and available resources (Bekele 2013; Nakashima et al. 2012). Local traditional leaders are the custodians of IK within communities and hence bear the potential to oversee its utilisation by villagers as part of their local area climate change adaptation initiatives.

As climate change continues to take its toll in many communities, conflicts in the wake of dwindling resources are also likely to mount as they are instigated by changes in habitat, movement of populations and adaptations to new types of economic systems (Bamidele 2011; Manyeruke, Hamauswa & Mhandara 2013; Mugandani et al. 2012). The long-known background of agroecological regions in Zimbabwe has changed over the years as a result of climate change with the main food producing regions II and III shrinking by 49% and 14%, respectively, while regions IV and V have extended by 5.6% and 22.6%, respectively, because of more aridity (Manyeruke et al. 2013). This shift in the pattern of climate has potential to trigger a lot of movement by people to find better living conditions, particularly with regard to smallholder farming. Field studies by Musarandega and Chingombe (2017) have revealed that many smallholder farmers from the drier western Chimanimani District are taking advantage of the Zimbabwean government land reform agenda and moving to the wetter eastern highlands, thus inversely rendering the once ecologically rich environment under extreme pressure. Such a move leads to overcrowding and mixing up of different family groups, which is a recipe for conflict of opinion and general social unrest in the district.

To date, conflicts over sharing resources within communities have widely been documented particularly with regard to water, which is directly affected by changing the climate (Manyeruke et al. 2013). Traditional leadership forms an immediate institution to resolve conflicts within communities because they are custodians of customary lands (Senganimalunje, Chirwa & Babalola 2012). According to the traditional conflict transformation concept (Boege 2006), the aim is to restore community order and harmony as opposed to punishment of perpetrators for deeds done in the past. The main emphasis is restitution as a basis for reconciliation within conflicting communities (Boege 2006). Thus, the ultimate goal is not to inflict punishment on perpetrators of conflict but to reintegrate them and restore good relations (ibid). External intervention alone to deal with issues of land and water shortage may be inadequate. Bamidele (2011) argues that when governments intervene and establish agricultural projects and commercial farm enterprises, for instance, the result is often further destabilisation of land relations. The traditional ‘restorative justice’ approach to justice as opposed to ‘western-style punitive justice’ (Boege 2006:07) is highly needed when dealing with environmental issues such as climate change. This is simply because social cohesion is a prerequisite to reducing the number of environmental deviants within communities.

For the purpose of cautiously mainstreaming traditional conflict transformation as mediation means, it is essential to consider five major weaknesses of traditional approaches as according to Boege (2006) that they:

- do not terminate violence in the long term
- often contradict universal standards of human rights
- have a limited sphere of applicability
- are geared towards the preservation of the ‘good old’ order
- are open to abuse. (p. 15)

The involvement of traditional authorities advanced by this paper finds support of scholars such as Marango (2017). Even in cases where government-instituted climate change strategies are in place, there are notable loopholes too. Manyeruke et al. (2013) argue that Zimbabwe’s agricultural policy proposed strategies meant to ensure food security in the country but the implementation of this policy has been marred by widespread socio-economic and political challenges. The strategies that the government adopted generally treated smallholder farmers as a homogenous group instead of considering their socio-economic and technological differences (Manyeruke et al., 2013). It is argued by Nakashima et al. (2012:10) that ‘some governmental policies have negative effects on adaptive capacity and by removing options and reducing choices, they constrain, restrict and undermine community efforts to adapt’.

It can still be further argued that even the efforts concerted on a global scale such as Kyoto Protocol to the UNFCCC of 1992 meant to cut down on GHG emissions face limitations as developed countries drag their feet in as far as implementation is concerned (Manyeruke et al., 2013). As alluded above, such shortfalls attached to traditional leadership by Boege (2006) can be dealt with through effective training of traditional authorities (Baldwin and Muyengwa 2014). Training helps traditional capacity building in terms of the law and its application. It enables them to respect the rights of the people that they administer and hence acts as a necessary prerequisite for justice to prevail in the adjudication of conflicts within communities. However, with regard to training, research has shown that traditional leaders learn better when other community members are also trained with them (Baldwin & Muyengwa 2014).

## Conclusion and recommendations

This review revealed that since time immemorial, communities have always been confronted with weather uncertainty and a host of related problems. In response to that, communities have always devised mechanisms to cope with related negative developments. Even before the advent of colonisation and western influence, LTAs have played a leading role in mobilising communities to cooperate in coping with weather-related eventualities. To date, traditional leaders still have the potential to harmonise the allocation and management of land and land-based natural resources. They are constitutionally recognised in the country and they fall under the local government ministry. The leadership comprises a tri-level hierarchy of traditional power involving chiefs, headmen and village heads.

Succession with regard to traditional leadership is on the hereditary basis as opposed to elected local government structures that are instituted on the basis of Acts of Parliament. Traditional leaders live in close proximity to their subjects and have more direct control on their subjects than outside government agents such as rural district councils. This structure gives them a vintage social position to oversee the mainstreaming of effective climate change adaptation practices within communities. Based on the findings of this review paper, the following recommendations are made:

- LTAs in Zimbabwe must be harnessed as a possible strategy of combating the negative effects of climate change.
- Researchers, scholars and development agencies must assist in the effort to complement traditional and modern climate change strategies in order to reduce the negative effects of climate change.
- Government agents such as police, rural district council officials, politicians and other outsiders must play a role in supporting the efforts of traditional in their execution of the *Traditional Leaders Act*.
